# Prevalence and Factors Associated with Metabolic Syndrome in Patients at a Psychosocial Care Center: A Cross-Sectional Study

**DOI:** 10.3390/ijerph191610203

**Published:** 2022-08-17

**Authors:** Dandara Almeida Reis da Silva, Ludmila Santana de Almeida, Livia Lugarinho Correa, Rodrigo Fernandes Weyll Pimentel, Antonio Marcos Tosoli Gomes, Ana Gabriela Travassos, Adriana Mattos Viana, Monique Magnavita Borba da Fonseca Cerqueira, Marcio Costa de Souza, Anderson Reis de Sousa, Paulo José Bastos Barbosa, Julita Maria Freitas Coelho, Lucelia Batista Neves Cunha Magalhães, Argemiro D’Oliveira Júnior, Jorge Lopes Cavalcante Neto, Charles Souza Santos, Luiz Carlos Moraes França, Juliana de Lima Brandão, Livia Fajin de Mello dos Santos, Helena Ferraz Gomes, Ellen Marcia Peres, Thais Regis Aranha Rossi, Kairo Silvestre Meneses Damasceno, Millena Conceição das Mercês, Sandra Lúcia Fernandes, Eline de Almeida Soriano, Isolda Prado de Negreiros Nogueira Maduro, Tatiana Santos Brandão, Amanda Cardoso Menezes, Amália Ivine Costa Santana, Magno Conceição das Merces

**Affiliations:** 1 Departamento de Ciências da Vida, Universidade do Estado da Bahia (UNEB), Salvador 41150-000, Bahia, Brazil; 2 Instituto Estadual de Diabetes e Endocrinologia Luiz Capriglione (IEDE), Rio de Janeiro 22451-000, Rio de Janeiro, Brazil; 3 Programa de Pós-Graduação em Ciências da Saúde, Universidade Federal da Bahia (UFBA), Salvador 40026-010, Bahia, Brazil; 4 Hospital Universitário Professor Edgard Santos (HUPES/UFBA), Universidade Federal da Bahia, Salvador 40150-000, Bahia, Brazil; 5 Centro Universitário UnidomPedro, Salvador 40010-020, Bahia, Brazil; 6 Faculdade de Enfermagem, Universidade do Estado do Rio de Janeiro (UERJ), Rio de Janeiro 20551-030, Rio de Janeiro, Brazil; 7 Centro Especializado em Diagnóstico, Assistência e Pesquisa (CEDAP), Secretaria de Saúde do Estado da Bahia, Salvador 40100-160, Bahia, Brazil; 8 Escola de Enfermagem, Universidade Federal da Bahia (UFBA), Salvador 40110-060, Bahia, Brazil; 9 Instituto Federal da Bahia (IFBA), Simões Filho 43700-000, Bahia, Brazil; 10 Faculdade de Tecnologia e Ciências (UNIFTC), Salvador 41741-590, Bahia, Brazil; 11 Universidade do Sudoeste da Bahia (UESB), Jequié 45200-000, Bahia, Brazil; 12 Associação Brasileira de Nutrologia (ABRAN), Catanduva 15801-150, São Paulo, Brazil; 13 Hospital Ana Nery (HAN), Salvador 40301-155, Bahia, Brazil

**Keywords:** metabolic syndrome, mental disorders, obesity, multimorbidity

## Abstract

Background: Metabolic syndrome (MS) is associated with greater risk of morbimortality and it has high prevalence in people with mental illness. Objective: Estimate the prevalence of Metabolic Syndrome (MS) and its associated factors in the patients of a Psychosocial Care Center (CAPS in Brazilian Portuguese) in the city of Salvador, state of Bahia, Brazil. Method: Cross-sectional study set at CAPS in the city of Salvador-Bahia between August 2019 and February 2020. MS was evaluated according to the National Cholesterol Education Program’s Adult Treatment Panel III. In addition to descriptive statistics, gross and adjusted prevalence ratios were described. Results: MS was found in 100 (35.2%) individuals, 116 (40.9%) were obese and 165 (58.1%) had increased waist circumference. Polypharmacy was identified in 63 (22.3%) patients and 243 (85.9%) used antipsychotics. Under gross evaluation, women (PR = 1.88; 95%CI: 1.35–2.63) and those who used antidepressants (PR = 1.41; 95%CI: 1.05–1.88) showed an association with MS. After logistic regression, depression (PR = 1.86; 95%CI: 1.38–2.51), acanthosis (PR = 1.50; 95%CI: 1.18–1.90), use of antipsychotics (PR = 1.88; 95%CI: 1.13–2.75), and hypertriglyceridemic waist (PR = 3.33; 95%CI: 2.48–4.46) were associated with MS. Conclusion: The prevalence of MS signals multimorbidity among individuals with mental disorders and suggests a need for clinical screening.

## 1. Introduction

Metabolic Syndrome (MS) consists of a confluence of metabolic changes related to insulin resistance, prothrombotic and inflammatory status. MS is associated with a greater chance of cardiovascular (CVD) outcome (RR 1.99 for myocardial infarction and a RR 2.4 for CVC mortality) and a 1.5-fold increase in all-cause mortality [1]. Individuals with MS can doubly develop diabetes mellitus [2], and are 1.6-fold (CI: 1.28–2.01) more likely to die from coronary etiology [3].

Although MS can consist of up to five determinants, such as elevation of triglycerides, increase of blood glucose and blood pressure, and reduced HDL-cholesterol levels, visceral adiposity increase, which is measured by the abdominal circumference, is the main marker of the syndrome [4]. In line with that main marker and consequent obesity, MS has reached larger portions of the population. According to data from the National Health and Nutrition Examination Survey, MS affected about 16% of non-obese Americans and 34.2% among all 51,000 American adults analyzed between 2007 and 2012 [5]. The Brazilian data are not different, and the prevalence of MS ranged from 22.7% [6] to 48.6% [7] in Brazilian groups, with a lower prevalence found in rural populations [8]. MS is associated with an increase in waist circumference and an alteration of the metabolic pattern, and is therefore related to obesity. It is mainly noted among women, individuals of older age, and people who are less formally educated [9]. In Brazil, per data from the Chronic Disease Surveillance by Telephone Survey (*Vigilância de Doenças Crônicas por Inquérito Telefônico—VIGITEL*), obesity has a prevalence similar to MS, with higher frequency among less formally educated individuals and among older age groups [10].

Furthermore, some groups appear to be at greater risk of MS, which is the case for people with mental disorders. In this regard, studies found a high prevalence of MS among individuals with mental illness, ranging from 29.4 to 67.9% [11,12], and a prevalence risk of 1.58 higher among mentally ill individuals in comparison to the general population [12]. More alarming data, such as the absence of dietary interventions or physical activity programs, were found in the entire sample, including glycemic alterations untreated in 63.9% and alterations in the lipid profile among 81.7% of the referred mentally ill group [10], which demonstrates neglect of other patients’ demands. In addition, there are few data in the Brazilian healthcare model focused on the transdisciplinary monitoring and social reintegration proposed by the Psychosocial Care Centers (*Centros de Atenção Psicossocial*—CAPS in Brazilian Portuguese). CAPS were designed as a new model of mental healthcare in Brazil, that is, an alternative to the hospitalization of patients with various psychiatric disorders. The CAPS health system consists of a minimum team of psychiatrists and nurses specializing in mental health, and it offers multidisciplinary care provided by professionals with higher education in the areas of health and physical education and by other health professionals without a mandatory superior in a non-hospital care regime, with group or individual activities, according to the patient’s demands. These centers are exclusively focused on mental healthcare, while other health monitoring needs are neglected.

Brazilian studies involving psychiatric patients and MS in an outpatient setting are scarce. Given this contextualization, it is still worth acknowledging the scarcity of Brazilian data or studies in the current field of mental health in Brazil. Thus, the objective of this study was to estimate the prevalence of Metabolic Syndrome (MS) and its associated factors in the patients of a Psychosocial Care Center in the city of Salvador, state of Bahia, Brazil.

## 2. Materials and Methods

This is a cross-sectional, descriptive, and exploratory study, carried out between August 2019 and February 2020 at a CAPS facility in the city of Salvador, Bahia, Brazil. A structured questionnaire was given to individuals undergoing treatment for psychiatric disorders, aged 18 years or older, who were attending for medical care on the day of the survey. The data collection procedures were performed by two trained researchers: an endocrinologist and a medical student. 

Pregnant women and patients with cirrhosis were excluded due to the increase of their abdominal areas, as well as were patients diagnosed with bulimia, anorexia, bigorexia, and drug addiction once they could have laboratory alterations along with MS. It should be noted that members of the latter group of patients were not treated in the CAPS facility that was included in the study. Patients with acute psychosis were also excluded due to the difficulty of performing analysis on such patients. 

The sample was delimited based on a sample calculation for a finite population by considering the number of 1006 active individuals in the unit, at an average of the three months before the study, and based on a Brazilian study [12] that found 29.4% prevalence of MS in people with mental illness during hospitalization. An absolute error of 5% and a confidence level of 95% were also considered. The value obtained through the sample calculation was 241, and 10% was added to suppress potential filling errors and losses, in addition to increasing the power of the study, by reaching 265 individuals. The calculation was performed by using the Epi Info 7.0 software (Centers for Disease Control and Prevention, Atlanta, GA, USA).

Sociodemographic data were obtained at the time of the interview. The psychiatric diagnoses were obtained from the available medical records of the patients who agreed to participate in the research. 

For the diagnosis of MS proposed by the National Cholesterol Education Program’s Adult Treatment Panel III report (NCEP-ATP III) revised in 2009 [4], at least three of the following five diagnostic criteria must be detected: triglycerides ≥ 150 mg/dL, blood glucose ≥ 100 mg/dL, HDL cholesterol < 50 mg/dL for women and <40 mg/dL for men, blood pressure ≥ 130 mmHg × 85 mmHg, and waist circumference (WC) ≥ 88 cm for women and ≥102 cm for men. It is worth noting that the diagnosis of arterial hypertension and/or diabetes from medications to treat these comorbidities were also considered since they do not invalidate the MS criteria. In addition, they can be used to replace the parameters used to measure blood pressure and fasting blood glucose [4]. 

Hypertriglyceridemic waist phenotype is defined by the concomitant elevation of serum triglyceride and WC levels. It was first described by Lemieux in 2000 [13]. Due to the lack of consensus in the literature on waist circumference cut-off points, the values adopted in this study are recommended by the NCEP, which are defined as inadequate when WC ≥ 102 cm for men and ≥88 cm for women. The value ≥ 150 mg/dL was defined as abnormal for triglycerides. 

Twelve hours of fasting was advised to collect laboratory tests. Enzymatic techniques were used to quantify the laboratory tests evaluated (fasting blood glucose, HDL cholesterol, and triglycerides) in serum plasma.

The clinical evaluation as well as the application of the questionnaires were carried out in an environment with privacy and during individual consultations performed by one of the two evaluators in the study.

The blood pressure measurement used was the average of the measurements obtained at the beginning and the end of the interview. The blood pressure was measured by Premium^®^ analog tensiometer with an appropriate cuff for the individual’s limb to calculate the average of the measurements obtained. The individual was to be sat with both feet on the floor for at least 5 min before the assessment.

The waist circumference was measured by using an ISP^®^ inelastic tape in an imaginary line in the middle-third between the antero-superior iliac crest and the last costal arch. To calculate the body mass index (BMI), weight and height were evaluated by using a Lider^®^ single scale, with a coupled stadiometer, model LD 1050, with 100 g precision, and weight variation between 2 and 300 kg. At the time of these measurements, the patient remained without shoes and excessive clothes. Obesity was defined as BMI ≥ 30 and overweight as BMI ≥ 25, according to WHO criteria [14].

Prescription data were reviewed for all patients, and medical records were evaluated by the medical researcher. The drugs considered were the ones prescribed in the consultation prior to the evaluation day of the research. Polypharmacy was defined as the use of five or more psychotropic drugs [15].

MS was considered as the only outcome variable. The following variables were considered as exposure: age, sex, self-reported race, education, polypharmacy, class of psychotropic drugs in use, psychiatric diagnosis, presence of acanthosis nigricans, and self-reported lifestyle (physical activity, alcohol, and tobacco consumption). Age was dichotomized according to the median line (abnormality under the Kolmogorov–Smirnov test), and eight years of education were defined as the education level cutoff point because this is the standard number of primary school years in Brazil. The variables related to life habits were noted as follows: self-reported consumption or absence of alcohol consumption was considered for smoking and alcohol consumption, regardless of the amount consumed daily, and physical activity was defined in two classes: absent/irregular and regular.

The Statistic Package for Social Sciences—SPSS software, version 22.0 (IBM, Armonk, NY, USA), for Windows was used for statistics analysis. Descriptive statistics was used to describe the population studied as well as to describe the estimate of MS prevalence. The following data were obtained: absolute and relative frequencies for categorical variables, measures of central tendency, and dispersion for describing the continuous variables. The description of the frequencies for psychiatric comorbidities was performed based on the distribution of each, between individuals with and without MS.

A bivariate analysis was then conducted to assess the gross association between independent and dependent variables (MS) by using Prevalence Ratios (PR), their respective 95% Confidence Intervals (CI), and a significance level of 5% (*p* value ≤ 0.05). The Pearson’s chi-square test was used for analyzing the statistical significance of the associations found and the selection to the next step. For bivariate analyses, continuous or polytomous independent variables were dichotomized.

For inclusion in the multivariate analysis, under the Backward logistic regression model, specialized literature and associations with a value of *p* ≤ 0.25 were considered. Afterwards, it was possible to estimate the factors associated with MS by using the value of *p* ≤ 0.05 as the selection criterion to keep the variable in the final model. The PR and respective CI were obtained through Poisson’s Robust Regression, which is a method also used by Coutinho, Scazufca, and Menezes [16], and Francisco et al. [17] for converting Odds Ratio (OR) (obtained in logistic regression models) into PR.

The following procedures and data were used to analyze the adequacy of the final regression model: Hosmer and Lemeshow goodness-of-fit, the area under the ROC curve, and the VIF (Variance Inflation Factor) Statistics Test. Based on this information, possible collinearities were identified between the variables and existence of influential observation patterns.

The research protocol was approved by the Ethics Committee of the State University of Bahia, under CAAE number 13159819.6.0000.0057. All participants signed the informed consent form or the term of acceptance when applicable.

## 3. Results

Three hundred forty-four (344) patients were approached, of whom 284 agreed to participate in the research. Out of these 284, 214 had laboratory tests. The clinical and social characterizations, in addition to the samples’ lifestyle habits, are described in Table 1. There was no statistically significant difference between individuals with and without adherence to laboratory tests when evaluated: anthropometric data, blood pressure levels, and sociodemographic data. Psychiatric characteristics, such as the profile of psychiatric diagnoses and polypharmacy, also showed *p* > 0.05 between the individuals who concluded and did not conclude the participation in the study. 

MS was identified in 100 individuals with a prevalence of 35.2% in the general sample and 46.5% among those with laboratory tests available. There was one death during the study period due to hypertriglyceridemia pancreatitis. The most common change was increased abdominal adiposity. One hundred and sixty-five (58.1%) individuals had increased waist circumference (83.2% of larger women WC greater than or equal to 88cm, while 27.9% of men had WC greater than or equal to 102 cm), which follows the trend of obesity in the studied group, as shown in Table 1. Thus, we identified that more than half of the population studied is overweight. Among the obese individuals, 15 (12.9%) had a BMI ≥ 40 kg/m^2^.

The other MS components presented the following frequencies: 137 (48.2%) individuals had fasting blood glucose ≥ 100 mg/dL, the systolic pressure was increased in 88 (31%) patients and the diastolic pressure in 106 (37.3%). Serum triglyceride levels were greater than 150 mg/dL in 98 (34.5%) individuals and 105 (37%) had triglyceride values below the target defined for MS (7.9% of men and 43.2% of the women).

The bivariate analysis between sociodemographic, clinical, and psychiatric data and MS are shown in Table 2 and Table 3.

In the evaluation of the mean blood pressure, 88 (31%) individuals had an increase in pressure compatible with the values defined as elevated by the NCEP criteria, however, only 58 (20.4%) individuals took any blood pressure control medicine. The same was noted for glycemic control, in which blood glucose levels equal to or greater than 100 mg/dL were found in 48 people. Twenty-seven (27) of them (56.3%) did not receive treatment for hyperglycemia. The mean value of glycemia was 104.4 ± 38.8 mg/dL and glycemia values greater than or equal to 126 mg/dL were found in twenty-nine individuals.

When carrying out the evaluation of the drugs used for the metabolic comorbidities that are defined as components of MS (dyslipidemia, hypertension, and diabetes), it was identified that five individuals used five or more drugs and all belonged to the group that completed the study. When the number of psychotropic and non-psychotropic drugs were added in the general sample (n: 284), 104 fulfilled the concept of polypharmacy when using five or more drugs, of which 34 (32.7%) were diagnosed with MS. Data regarding the use of psychotropic drugs are presented in Table 1 and Table 3. There was no psychiatric diagnosis in the medical records for 15 (5.3%) patients, and it was not possible to recover these data from two (0.7%) individuals. The most prevalent psychiatric comorbidity was schizophrenia, which affected 163 (50.7%) individuals, followed by depressive disorders 57 (20.2%). Among the other disorders not described in Table 2, there were some with a frequency less than 5% of the sample, such as unspecified mood disorder, personality disorder, attention deficit hyperactivity disorder, dissociative disorder, and simulation. Another group not mentioned had so-called organic disorders, as they correspond to a wide range of pathologies, and were diagnosed in 29 (10.3%) individuals. Among patients with multiple psychiatric comorbidities, 10 (3.5%) have three psychiatric diagnoses. There was no record of more than three psychiatric diagnoses in the same individual.

Antipsychotics and benzodiazepines were the most used psychotropic drugs in 243 (85.9%) and 157 (55.5%) of the prescriptions, respectively. Polypharmacy was identified in 63 (22.3%) patients. 

The gross prevalence ratio showed an association between the variable ‘women,’ the presence of acanthosis nigricans, changes in the hypertriglyceridemic waist, diagnosis of depression, and consumption of antidepressants as factors associated with the diagnosis of MS.

In the logistic regression, the variables are as follows: acanthosis (adjusted PR: 1.50), antipsychotic (adjusted PR: 1.76), depression (adjusted PR: 1.86) and hypertriglyceridemic waist (adjusted PR: 3.33). These variables were statistically associated with MS. It is noteworthy that the final model exhibited the ROC Curve = 0.87 and Hosmer’s test/Lemeshow’s goodness of fit = 0.41, indicating adequate discrimination power and well adjustment to the data. No collinearity was found between the variables (VIF < 5), and patterns of influential observations were not identified (Table 4).

## 4. Discussion

According to the national and international literature investigated to date, this is the first study to assess the prevalence and factors associated with MS among individuals monitored by CAPS.

In this study, the high prevalence of MS and overweight/obesity status were generally found among individuals with mental illness, and those with depression were the most affected, with a prevalence ratio of 1.86 (95% CI 1.38–2.51). In addition to representing the most prescribed class of psychotropic drugs, antipsychotics were the only ones listed in the final model associated with MS (PR: 1.76; 95% CI: 1.13–2.75, respectively). Despite not being kept in the multivariate analysis, women showed an association with the diagnosis of MS in the bivariate analysis (PR: 1.88; 95% CI: 1.35–2.63). No association was found between MS and the age of the individuals studied in the assessment of gross and adjusted PR.

Despite the prevalence of MS being found to be higher than that described in the baseline study by Rocha et al. (29.4%) [12], other groups had similar and even higher prevalence of MS among individuals with mental illness [18,19]. In a systematic review of general population studies in Brazil published in 2013, an average prevalence of 29.6% was found [8]. However, similarly to the whole world, we noticed an increase in the frequency of obesity in the Brazilian population, reaching 19.8% of adults in the last survey published in 2019 [10], which likely contributes to the increase in morbidity caused by MS. Such analysis reinforces the findings in this study, where there is an increase of MS frequency compared to the study by Rocha et al. [12].

Additionally, the described high prevalence of MS, the prevalence of obesity (40.9%), and the abdominal adiposity deposition (58.1%) found are reasons to call our attention to the possibility of greater metabolic illness in these individuals. Despite the data in Brazil and worldwide showing an increase in the prevalence of obesity [10,20] and abdominal adiposity [21], the data from the present study exceeds the prevalence of other local studies with populations from geographically similar regions, which describe the prevalence of obesity and overweight status as 23.5% and 42.9%, respectively [22]. Furthermore, in other studies developed by this research group, increased abdominal adiposity was found in 41.5% of nursing professionals, and ranged from 34.8% among men to 38.8% of women physicians [23,24]. Such a condition may contribute to premature mortality in this group, which is already described as having a reduced life expectancy [25], and who reach up to twelve years old when they have schizophrenia [26].

Similarly, other studies have also been unable to identify an association between MS and schizophrenia [18]. However, the association between MS and depression is dangerous and should serve as a warning because it can generate a combination of risk factors for mobility impairment. Depression has been described as a cardiovascular risk factor, a cause for the loss of active years, and has been associated with MS [27,28].

The use of antipsychotics, as well as the findings of a previous study, were related to a higher prevalence of MS, suggesting that greater attention that should be paid to individuals who use this class of drugs [18]. Given this scenario of such a prescribed class, this study highlights the need for reflection regarding the need to minimize these metabolic changes, and consequently, cardiovascular changes.

Certain incentives are important, such as encouraging non-pharmacological measures for metabolic control and prevention, as well as promoting healthy eating and practicing physical activity often. Non-pharmacological intervention studies focused on improving metabolic performance and reducing obesity have been developed [29], including strategies regarding the use of remote technology [30], but the existing data are conflicting [31,32], and there are reduced cost-effectiveness descriptions for interventions promoted by a lifestyle program within a mentally ill population in Canada [33].

Screening measures are easily applied by health service professionals, and they are necessary for optimizing the flow of referrals for metabolic assessment of individuals at metabolic risk. Notably, 56.2% of medical service patients do not undergo medical monitoring, nor are they evaluated by a psychiatrist during service. This therapeutic inertia has already been described by other groups [34,35].

The association between MS and acanthosis has also been described by Mercês et al. [23] in a population in northeastern Brazil. Although still controversial in the literature, the hypertriglyceridemic waist phenotype has been studied as a possible simplified marker of cardiovascular risk [36], and is associated with the development of diabetes mellitus [37]. Based on the Kappa index, the hypertriglyceridemic waist correlated to cardiovascular risk to diagnose MS ranged from 0.42 to 0.58 [38].

An unexpected finding in this study was the non-association of MS in the final model related to the variable ‘age’. The literature already found this non-association, regardless the presence of a mental disorder [39]. This study could reinforce this point based on the young age of the studied group.

The high prevalence of MS and obesity, as previously described, should be an alert for better clinical monitoring of individuals with mental illness, so this study demonstrates its relevance to the promotion of a local diagnosis of these individuals’ metabolic illness, which is reinforced by the literature [18,19]. Thus, the occurrence of MS is a marker of multimorbidity, which, despite being a recent concept, has been described as more prevalent among individuals with mental illness compared to the general population [40], and it is associated with polypharmacy and worse outcomes in the population with mental illness [41].

However, we need to reflect on the limitations of this study: (I) impossibility of defining causality because only association analyzes can be established; (II) high frequency of non-adherence to laboratory tests can have an impact on the evaluation of the data presented, (III) obtaining information on self-reported lifestyle habits also has limitations, which need to be assessed considering the peculiarities of the participants in the study. In view of the limitations mentioned, the methodological robustness used in the present study stands out.

## 5. Conclusions

The high frequency of obesity and metabolic syndrome is cause for increased alertness for multimorbidity in this group, and prompts reflection on the importance of basic assessment for metabolic changes among individuals with mental illness in order to provide integrated care and optimize their quality of life and lifespan. Depression, the use of antipsychotics, as well as the acanthosis nigricans, which are clinically and easily obtained data, are proven to be risk factors for MS. 

The data warn of the need to update the healthcare team to detect individuals at higher risk for metabolic diseases, as well as the need for an expanded medical evaluation, with the participation of the clinical physician, in caring for individuals with mental illness. In this scenario, anthropometric assessment and the training to recognize acanthosis nigricans can be combined to screen individuals with higher metabolic risk by using fewer complex features and at a lower cost. Encouraging adherence to a change in lifestyle with regular physical activity and healthy eating is mandatory for those with mental illness as a way to improve their quality of life and to reduce obesity and the risk of metabolic illness.

## Figures and Tables

**Table 1:** General description, by sex, of the total users of a Psychosocial Care Center, who agreed to participate in the study, Salvador, Bahia, Brazil, 2019 (n = 284).

Variables	Description	
	Men (129)	Women (155)
**Sociodemographic**		
Age (years) (n = 284)		
Mean ± SD	42.6 ± 11.6	45.8 ± 12.0
Median (IQ)	41 (34–51)	44 (36–55)
Self-reported race n (%) (n = 284)		
White	11 (8.5)	14 (9.0)
Black	45 (34.9)	50 (32.3)
Brown	70 (54.3)	84 (54.2)
Others	3 (2.3)	7 (4.5)
Education n (%) (n = 282) ᵃ		
No schooling	6 (4.7)	19 (12.3)
1–7 years	60 (46.5)	46 (29.7)
8–12 years	57 (44.3)	74 (47.8)
≥12 years	5 (3.9)	15 (9.7)
Marital status n (%) (n = 284)		
Married/common-law marriage	28 (21.7)	37 (23.9)
Single	95 (73.6)	90 (58.1)
Widowed/divorced	6 (4.7)	28 (18.0)
**Life Habits**		
Smoking n (%) (n = 284)		
No	99 (76.7)	133 (85.8)
Yes	30 (23.3)	22 (14.2)
Alcohol consumption n (%) (n = 284)		
No	117 (90.1)	144 (92.9)
Yes	12 (9.3)	11 (7.1)
Regular practice of physical activity n (%) (n = 284)		
Regularly	44 (34.1)	45 (29.9)
Sedentary	85 (65.9)	110 (71.1)
**Clinical**		
Weight (kg) (n = 284)		
Mean ± SD	78.1 ± 16.6	78.1 ± 17.1
Median (IQ)	76.5 (65.9–88.8)	76.1 (65.6–89.4)
BMI (kg/m^2^) (n = 284)		
Mean ± SD	26.5 ± 4.7	31.4 ± 6.3
Median (IQ)	26.0 (23.3–29.9)	30.8 (26.9–36.5)
BMI ≥ 25 n (%)	76 (58.9)	130 (83.9)
BMI ≥ 30 n (%)	30 (23.3)	86 (52.9)
Waist (cm) (n = 284)		
Mean ± SD	94.3 ± 13.4	100.6 ± 14.1
Treatment for Diabetes Mellitus n (%) (n = 284)		
No	116 (89.9)	132 (85.2)
Yes	13 (10.1)	23 (14.8)
Treatment for Arterial Hypertension n (%) (n = 284)		
No	110 (85.3)	116 (74.8)
Yes	19 (14.7)	39 (25.2)
Treatment for Dyslipidemia n (%) (n = 284)		
No	118 (91.5)	135 (87.1)
Yes	11 (8.5)	20 (12.9)
Presence of acanthosis nigricans n (%) (n = 276) ᵃ		
No	115 (89.2)	105 (67.7)
Yes	11 (10.8)	45 (32.3)
Hypertriglyceridemic waist n (%) (n = 213) ᵃ		
No	76 (58.9)	67 (43.2)
Yes	19 (41.1)	51 (56.8)
Number of psychiatric drugs in use n (%) (n = 283) ᵃ		
Mean ± SD	3.7 ± 0.1	3.6 ± 0.1
Medical follow-up outside the psychiatric service n (%) (n = 260) ᵃ		
No	81 (63.3)	65 (41.9)
Yes	34 (36.7)	80 (58.1)

^a^ variable with missing data; SD: standard deviation; IQ: interquartile range; BMI: body mass index.

**Table 2:** Gross prevalence ratio of metabolic syndrome and its 95% confidence intervals according to psychiatric diagnosis and use of psychiatric drugs in the users of a Psychosocial Care Center, Bahia, Brazil, 2019 (n = 215).

	Metabolic Syndrome		
Variables	P (%) ^b^	PR ^c^ (CI 95%) ^d^	*p*-Value ^e^
**Use of psychotropic drugs by class (n = 215)**			
Antipsychotics			
No	9 (33.3)	1.00	
Yes	91 (48.4)	1.45 (0.83–2.52)	0.14
Antidepressants			
No	45 (39.1)	1.00	
Yes	55 (55.0)	1.41 (1.05–1.88)	0.02 *
Mood stabilizers			
No	55 (43.3)	1.00	
Yes	45 (51.1)	1.18 (0.88–1.57)	0.26
Benzodiazepines			
No	55 (43.3)	1.00	
Yes	45 (51.1)	1.06 (0.79–1.41)	0.69
**Psychiatric diagnoses (n = 213) ᵃ**			
Schizophrenia			
No	66 (50.4)	1.00	
Yes	34 (40.5)	0.80 (0.59–1.09)	0.16
Depression			
No	74 (42.8)	1.00	
Yes	26 (61.9)	1.45 (1.07–1.94)	0.03 *
Anxiety disorders			
No	94 (46.5)	1.00	
Yes	6 (46.2)	0.99 (0.54–1.82)	0.98
Bipolar disorder			
No	82 (45.6)	1.00	
Yes	18 (51.4)	1.12 (0.79–1.62)	0.52
Intellectual Disability			
No	84 (46.4)	1.00	
Yes	16 (47.1)	1.01 (0.69–1.49)	0.94
**Multiple Psychiatric Diagnosis (n = 215) ᵃ**			
No	71 (33.0)	1.00	
Yes	29 (13.5)	0.92 (0.67–1.27)	0.61

^a^ variable with missing data; ^b^ P: prevalence of the outcome between exposed and unexposed; ^c^ PR: gross prevalence ratio; ^d^: 95% confidence intervals; ^e^ Pearson’s chi-square test; * statistical significance.

**Table 3:** Gross prevalence ratio of metabolic syndrome and its 95% confidence intervals according to sociodemographic and clinical variables in the users of a Psychosocial Care Center, Bahia, Brazil, 2019 (n = 215).

	Metabolic Syndrome		
Variables	P (%) ^b^	PR ^c^ (CI 95%) ^d^	*p*-Value ^e^
**Sociodemographic**			
Sex (n = 215)			
Men	30 (31.3)	1.00	
Women	70 (58.8)	1.88 (1.35–2.63)	<0.01 *
Age (years) (n = 215)			
<43 years	42 (48.0)	1.00	
≥43 years	58 (58.0)	1.24 (0.93–1.67)	0.14
Self-referred race (n = 215)			
White	10 (55.6)	1.00	
Non-white	90 (45.7)	0.82 (0.53–1.27)	0.42
Education (n = 215)			
≥8 years	55 (47.8)	1.00	
<8 years	45 (45.0)	0.94 (0.70–1.26)	0.68
Marital status n (%) (n = 215)			
Married/common-law marriage Without partner	72 (44.2) 28 (53.8)	1.00 0.82 (0.60–1.11)	0.22
**Life Habits**			
Smoking n (%) (n = 215)			
No	78 (44.6)	1.00	
Yes	22 (55.0)	1.23 (0.89–1.71)	0.23
Alcohol consumption n (%) (n = 215)			
No	94 (47.7)	1.00	
Yes	6 (33.3)	0.69 (0.35–1.36)	0.24
Regular practice of physical activity n (%) (n = 215)			
Regularly	25 (39.7)	1.00	
Sedentary	75 (49.3)	1.24 (0.88–1.75)	0.19
**Clinical**			
Presence of acanthosis nigricans n (%) (n = 210) ᵃ			
No	64 (38.8)	1.00	
Yes	34 (73.9)	1.90 (1.47–2.46)	<0.01 *
Medical follow-up outside the psychiatric service n (%) (n = 198) ᵃ			
Yes	45 (46.7)	1.0	
No	48 (45.7)	1.06 (0.79–1.44)	0.65
Hypertriglyceridemic waist n (%) (n = 213) ᵃ			
No	36 (25.2)	1.00	
Yes	62 (88.6)	3.52 (2.62–4.72)	<0.01 *
Polypharmacy ^$^ (n = 215) ᵃ			
No	74 (44.3)	1.00	
Yes	26 (54.2)	1.22 (0.89–1.66)	0.23

^a^ Variable with missing data; ^$^ Only for psychotropics; ^b^ P: prevalence of the outcome between exposed and unexposed; ^c^ PR: gross prevalence ratio; ^d^: 95% confidence intervals; ^e^ Pearson’s chi-square test; * statistical significance.

**Table 4:** Factors associated with MS and obtained through multivariate analysis among the users of a CAPS.

Factors Associated with Metabolic Syndrome	PRadjusted	CI (95%)
Acanthosis Nigricans	1.50	1.18–1.90
Antipsychotics	1.76	1.13–2.75
Depression	1.86	1.38–2.51
Hypertriglyceridemic waist	3.33	2.48–4.46
Area under the ROC curve	0.87	
^¥^ Goodness-of-fit Test	0.41	

^¥^ Hosmer-Lemershow.
